# Comparison of Molecular and Parasitological Methods for Diagnosis of Human Trichostrongylosis

**DOI:** 10.3389/fcimb.2021.759396

**Published:** 2021-10-13

**Authors:** Mehdi Pandi, Meysam Sharifdini, Keyhan Ashrafi, Zahra Atrkar Roushan, Behnaz Rahmati, Nayereh Hajipour

**Affiliations:** ^1^ Department of Medical Parasitology and Mycology, School of Medicine, Guilan University of Medical Sciences, Rasht, Iran; ^2^ Department of Biostatistics, School of Medicine, Guilan University of Medical Sciences, Rasht, Iran; ^3^ Department of Microbiology, School of Medicine, Guilan University of Medical Sciences, Rasht, Iran

**Keywords:** human trichostrongyliasis, wet mount, Harada–Mori culture, Willis, agar plate culture, formalin ethyl acetate concentration, PCR

## Abstract

Human trichostrongyliasis is a zoonotic disease that is prevalent among rural populations in some countries. This study was performed to evaluate various parasitological methods and polymerase chain reaction (PCR) for the diagnosis of human trichostrongyliasis. A total of 206 fresh stool samples were collected from residents of endemic villages of Northern Iran. All samples were examined using conventional parasitological methods, including wet mount, formalin ethyl acetate concentration (FEAC), agar plate culture (APC), Harada–Mori culture (HMC), and Willis, along with the PCR technique. Among the total of 206 individuals examined, 72 people (35%) were found infected with *Trichostrongylus* species using combined parasitological methods. By considering the combined results of parasitological methods as the diagnostic gold standard, the Willis technique had a sensitivity of 91.7% compared with 52.8% for the APC, 40.3% for the HMC, 37.5% for FEAC, and 5.6% for the wet mount technique. The diagnostic specificity of all the parasitological methods was 100%. Furthermore, the PCR method detected *Trichostrongylus* spp. DNA in 79 fecal samples (38.3%) with a sensitivity of 97.2% and a specificity of 93.3%. According to the current findings, the Willis method was more sensitive than are the other parasitological methods in the diagnosis of human trichostrongyliasis. However, the PCR assay was more sensitive and more reliable in the detection of human trichostrongyliasis in comparison with the parasitological methods.

## Introduction

Nematodes of the genus *Trichostrongylus* are primarily parasites of herbivorous animals with a worldwide distribution. Ruminants are considered the most important reservoir for human trichostrongylosis (Ghadirian and Arfaa, 1975). Human infections associated with *Trichostrongylus* species have been reported sporadically from various countries of the Middle and the Far East, Africa, South America, Europe, and Oceania, with the highest prevalence rates reported in Iran (Ghadirian and Arfaa, 1975; Cancrini et al., 1982; Boreham et al., 1995; Lattes et al., 2011; Sato et al., 2011; Wall et al., 2011; Phosuk et al., 2013; Watthanakulpanich et al., 2013). In recent decades, a sharply decreasing trend was observed in the prevalence of most human soil-transmitted helminths (STHs) in Iran (Rokni, 2008; Sharifdini et al., 2017b; Sharifdini et al., 2020). However, recent epidemiological studies have demonstrated that trichostrongylosis is still a common helminth infection in humans in some parts of Iran, which results from its ability of zoonotic transmission to humans (Ashrafi et al., 2015; Gholami et al., 2015; Sharifdini et al., 2017a; Sharifdini et al., 2017c; Ashrafi et al., 2020).

Twelve valid species of *Trichostrongylus* have been detected from humans in various areas of the world, nine of which were only reported from Iran (Ghadirian et al., 1974; Ghadirian and Arfaa, 1975; Ghadirian, 1977; Sharifdini et al., 2017a). Within the past decades, in most parts of Iran, the predominant species of *Trichostrongylus* in humans were *Trichostrongylus orientalis* and *Trichostrongylus colubriformis* (Ghadirian and Arfaa, 1975). Agricultural use of night soil as fertilizer was an important reason for the high prevalence of *T. orientalis* in these regions because the transmission of this species primarily occurs from human to human (Ghadirian and Arfaa, 1975). At present, the predominant species is *T. colubriformis* because of its high prevalence in domestic animals and its high zoonotic potential (Gholami et al., 2015; Sharifdini et al., 2017a; Sharifdini et al., 2017c).

The transmission route of human infections is mainly through the ingestion of vegetables contaminated with filariform larvae (Roberts and Janovy, 2012). Although trichostrongylosis is generally considered asymptomatic and the only present finding is low-grade peripheral eosinophilia, heavy infections may be followed by abdominal discomfort, diarrhea, nausea, anorexia, weakness, flatulence, dizziness, generalized fatigue or malaise, and mild anemia (Ghanbarzadeh et al., 2019).

Definitive diagnosis of trichostrongylosis depends on observing the characteristic eggs in stool samples or finding the larva in fecal cultures (Roberts and Janovy, 2012). It should be noted that *Trichostrongylus* eggs may be mistaken with those of hookworms (Sato et al., 2011). Additionally, *Trichostrongylus* larvae are relatively similar to those of hookworms and *Strongyloides stercoralis*, which may be difficult to distinguish clearly (Roberts and Janovy, 2012). Although there are several parasitological methods for the detection of *Trichostrongylus* infection, there are limited studies that show comparisons of their sensitivity and specificity (Najmi et al., 2017; Saraei et al., 2019). Recently, only a few studies applied polymerase chain reaction (PCR)-based techniques for specific detection of *Trichostrongylus* spp. in human fecal samples (Gholami et al., 2015; Sharifdini et al., 2017c; Perandin et al., 2018). However, these studies had small sample sizes and insufficient power to evaluate their efficacy. In this study, we compared several parasitological methods along with conventional PCR for the diagnosis of *Trichostrongylus* infection in human fecal samples.

## Methods

### Study Area and Sample Size Determination

The study area comprises highly endemic villages of trichostrongylosis within the Fouman District in Guilan Province, Northern Iran. The sample size was calculated based on the prevalence of *Trichostrongylus* in the region using the following formula: *Z* ^2^ se (1 − se)/*d* ^2^ × prev, where prev is the prevalence of *Trichostrongylus*, se is sensitivity, *d* is the precision of the estimate, and *Z* is the standard score corresponding to 1.96. The prevalence rate of *Trichostrongylus* in the study area based on a pilot study and unpublished data was about 36%. For the calculation, a 95% sensitivity and a 5% precision of estimate were used. This gave a sample size of 197. To minimize errors arising from the likelihood of non-compliance, 5% of the sample size was added, giving a final sample size of about 206.

### Sample Collection

A total of 206 fresh stool samples were collected from the residents of the villages from June to October 2020. Fecal samples were transferred immediately after collection to the Department of Parasitology and Mycology, Guilan University of Medical Sciences.

### Parasitological Methods

All stool samples were examined using the wet mount, formalin ethyl acetate concentration (FEAC), agar plate culture (APC), Harada–Mori culture (HMC), and Willis techniques.

The APC method was applied for the detection of *Trichostrongylus* spp. larvae. In brief, 3–4 g of each fecal sample was placed on nutrient agar culture. After incubation for 3–5 days at room temperature (25–35°C), the plates were examined under a stereomicroscope for the presence of moving larvae or their tracks. In order to collect the larvae, the surface of the positive agar plates was washed out by lukewarm phosphate-buffered saline (PBS) solution. Larvae of *Trichostrongylus* species were identified from other probable intestinal nematodes, such as *S. stercoralis* and hookworms, based on morphological characteristics (Sharifdini et al., 2017a).

In the HMC method, approximately 2 g of each fresh stool sample was smeared on a folded strip of filter paper. After adding up to 5 ml of distilled water into a 15-ml falcon tube, the strip containing the fecal sample was placed into the tube and stored at room temperature for 7 days. Finally, the tube fluid was checked for detection of larvae of *Trichostrongylus* species (Harada and Mori, 1955).

For the sodium chloride flotation technique or the Willis method, about 2–3 g of each fecal sample was diluted in 20 ml of saturated salt solution (NaCl, 1.20 g/ml). The mixture was then filtered through sterile gauze and immediately transferred into a test tube. Then, a coverslip was placed carefully on top of the tube. After 15 min, the coverslip was lifted off the test tube and deposited on a microscope slide (Willis, 1921).

### Molecular Methods

#### DNA Extraction

For DNA isolation, about 2 g of each stool sample was processed using the sodium chloride flotation technique. Next, the supernatant was washed twice with distilled water, followed by centrifugation at 8,000 × *g* for 5 min to remove the salt. Subsequently, genomic DNA was extracted from the sediment using a commercial DNA extraction kit (Viragene, Tehran, Iran) based on the instructions on the manual and stored at −20°C for PCR amplification.

#### PCR Amplification

PCR reactions were performed in 20 μl volumes containing 2× red PCR premix (Ampliqon, Odense, Denmark), 20 pmol of each primer, and 3 μl of extracted DNA. The ribosomal DNA internal transcribed spacer 2 (*ITS*2) was amplified using forward primer (Tri-F: 5′-AATGAATTTCTACAGTGTGG-3′) and reverse primer (Tri-R: 5′-CATACATGTCCCTGTTTAAATC-3′), resulting in an amplicon size for *Trichostrongylus* spp. of 211 bp (Mizani et al., 2017). The PCR conditions comprised an initial denaturing step of 95°C for 5 min, followed by 35 cycles of denaturation at 95°C for 45 s, annealing at 54°C for 45 s, and extension at 72°C for 60 s, and a final extension at 72°C for 7 min. Finally, the PCR product was electrophoresed on 1.5% agarose gel and visualized with a UV transilluminator. Later, the PCR products were sent to a domestic sequencing company (Codon Genetic Company, Tehran, Iran) for sequence determination *via* the Sanger method.

To confirm the results of the molecular method, eight positive products—four positive and four negative parasitological stool samples—were selected randomly and sent to a domestic sequencing company (Codon Genetic Company, Tehran, Iran) for sequence determination via the Sanger method. Sequence results were manually edited and analyzed using Chromas (version 2.6) software. The sequences were compared with those submitted to GenBank using the BLAST system (http://www.ncbi.nlm.nih.gov/), and multiple sequence alignment was carried out using the Clustal W method of Bioedit software (version 7.2).

### Analytical Sensitivity and Specificity of PCR

To determine the analytical sensitivity of the molecular method, genomic DNA was extracted from 100, 50, 20, 10, 5, and from 1 egg of *Trichostrongylus* spp. using a DNA extraction kit (Viragene, Tehran, Iran) according to the manufacturer’s protocol. Subsequently, PCR for these samples was carried out as mentioned above.

The specificity of the PCR method was assessed using extracted DNAs from adult *Necator americanus*, *Taenia saginata*, *Haemonchus contortus*, *Marshallagia marshalli*, *Ostertagia ostertagi*, and *Rhabditis axei*, and also from stool samples infected with *S. stercoralis*, *Fasciola hepatica*, *Dicrocoelium denriticum*, *Cryptosporidium* sp., *Enterocytozoon bieneusi*, *Entamoeba coli*, *Giardia lamblia*, and *Blastocystis hominis*.

### Data Analysis

Data were analyzed using SPSS software (version 18, SPSS Inc., Chicago, IL, USA) to determine the diagnostic sensitivity and specificity of the molecular and parasitological methods.

### Ethical Approval

This study was reviewed and approved by the Ethics Committees of Guilan University of Medical Sciences, Iran (ref. no. IR.GUMS.REC.1398.434).

## Results

### Comparison of Parasitological and PCR Methods

**Figure 1:**
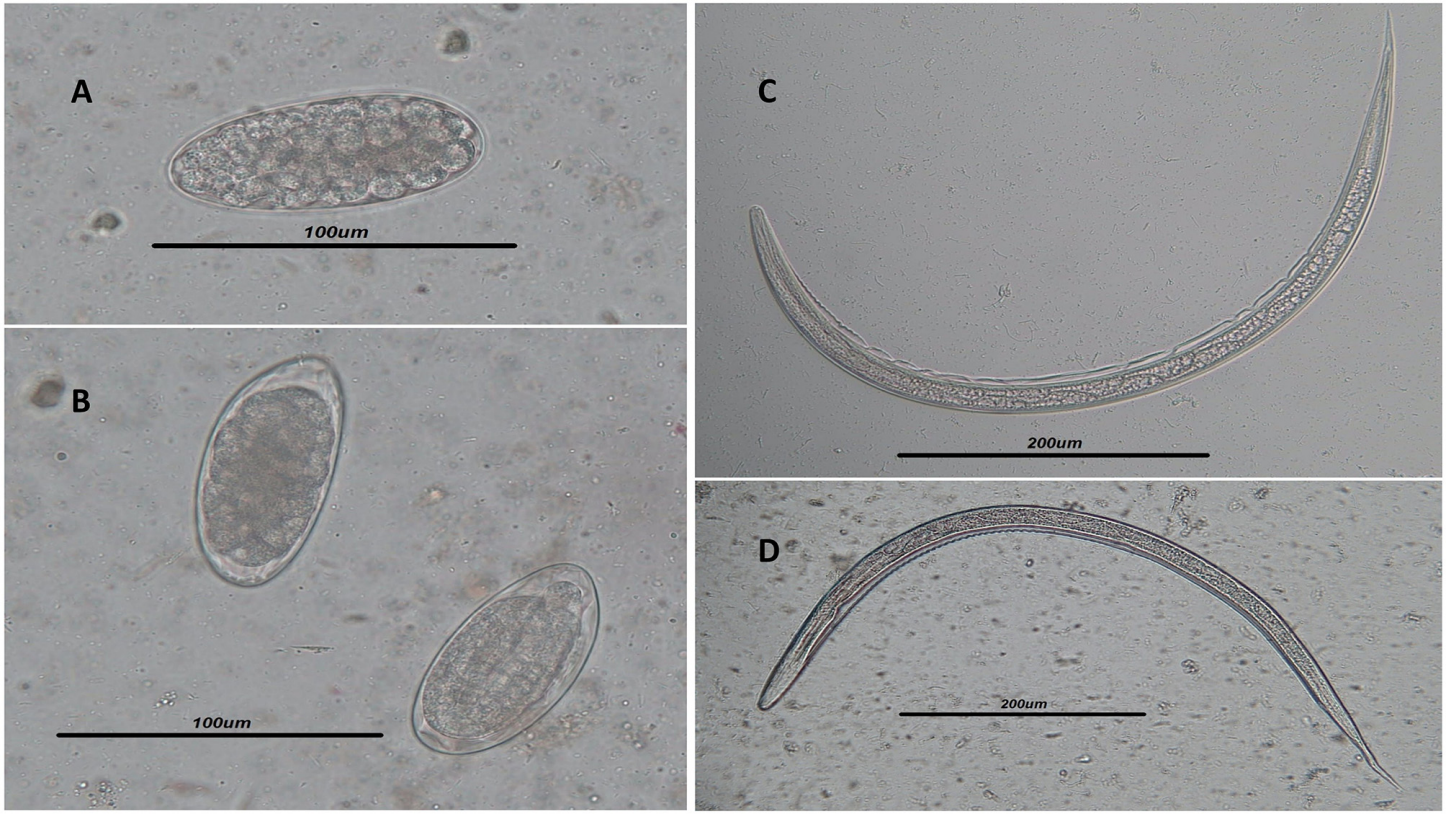
**(A, B)** Light microscope view of *Trichostrongylus* spp. eggs isolated by the Willis technique in the stool samples of infected humans. *Scale bar*, 100 μm. **(C, D)** Filariform larvae of *Trichostrongylus* spp. isolated from agar plate culture. *Scale bar*, 200 μm.

Among the total of 206 individuals examined, 72 people (35%) were found infected with *Trichostrongylus* species using combined parasitological methods (**Figure 1**). The detection rates for the wet mount, FEAC, APC, HMC, and Willis techniques solely were 4 (1.9%), 27 (13.1%), 38 (18.4%), 29 (14.1%), and 66 (32%), respectively (**Table 1**). The Willis method, as the most sensitive among the parasitological methods, could detect 4 of the 4 positive cases found using wet mount, 26 of the 27 by FEAC, 27 of the 29 by HMC, and 35 of the 38 by APC. In addition, the Willis technique detected 62, 40, 39, and 31 more samples, which were negative by wet mount, FEAC, HMC, and APC, respectively. Moreover, the other intestinal parasites detected in the current study using parasitological methods were *S. stercoralis* (0.97%), *G. lamblia* (1.4%), *B. hominis* (0.97%), and *F. hepatica* (0.48%).

**Table 1:** Comparison of the parasitological and molecular methods in the detection of *Trichostrongylus* spp. in fecal samples (*n* = 206).

	Wet mount	FEAC	APC	HMC	Willis	PCR
**Positive (72)**	4	27	38	29	66	79
**Negative (134)**	202	179	168	177	140	127
**Sensitivity (%)**	5.6	37.5	52.8	40.3	91.7	97.2
**Specificity (%)**	100	100	100	100	100	93.3

FEAC, formalin ethyl acetate concentration; APC, agar plate culture; HMC, Harada–Mori culture.

**Figure 2:**
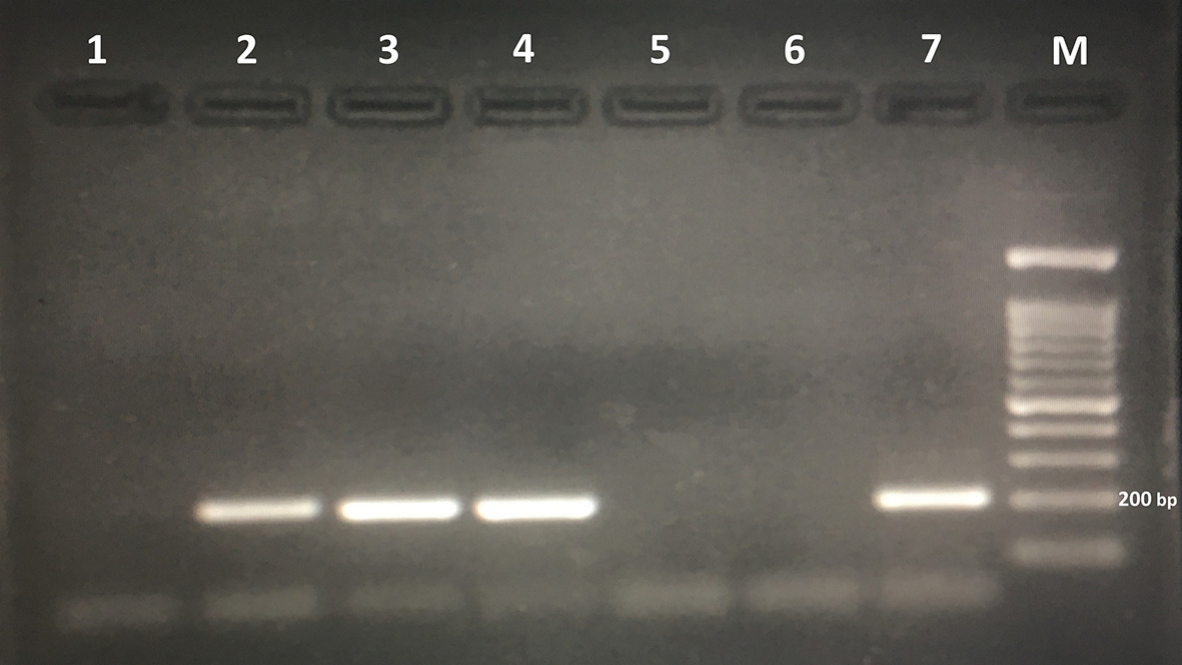
Agarose gel electrophoresis of polymerase chain reaction products amplified with genomic DNA from stool samples. *Lanes 2*, *3*, and *4*: polymerase chain reaction products of stool samples positive for *Trichostrongylus* spp. *Lane 6*: negative control. *Lane 7*: positive control (*Trichostrongylus colubriformis*). *M*, 100-bp DNA marker.

The PCR method detected *Trichostrongylus* species DNA in 79 out of 206 stool samples (38.3%) (**Figure 2**). All these positive cases were detected using wet mount, FEAC, and HMC. However, PCR failed to detect one APC and two Willis positive cases. Also, the PCR assay detected nine (4.37%) more samples, which were negative using the parasitological methods (**Table 2**). Sequence analysis of 167 bp of the eight randomly selected PCR products revealed that all of them had 100% similarity to *T. colubriformis* in the GenBank reference sequences. All positive isolates were registered in the GenBank database with accession numbers MW680815–MW680822.

**Table 2:** Evaluation of the PCR assay in comparison with parasitological methods for the diagnosis of *Trichostrongylus* spp. infection in stool samples (*n* = 206).

Methods		Wet mount		FEAC		APC		HMC		Willis	
		Positive (4)	Negative (202)	Positive (27)	Negative (179)	Positive (38)	Negative (168)	Positive (29)	Negative (177)	Positive (66)	Negative (140)
PCR	Positive (79)	4	75	27	52	37	42	29	50	64	15
	Negative (127)	0	127	0	127	1	126	0	127	2	125

FEAC, formalin ethyl acetate concentration; APC, agar plate culture; HMC, Harada–Mori culture

Since there is no valid gold standard for the diagnosis of *Trichostrongylus* spp. infections, the combined results of the parasitological methods were considered as the gold standard in this study. Therefore, the diagnostic sensitivity values of the wet mount, HMC, Willis, APC, FEAC, and PCR methods were calculated as 5.6%, 40.3%, 91.7%, 52.8%, 37.5%, and 97.2%, respectively. The diagnostic specificity of all the parasitological methods was 100%, while that of the PCR assay was 93.3%.

### Analytical Sensitivity and Specificity of PCR

**Figure 3:**
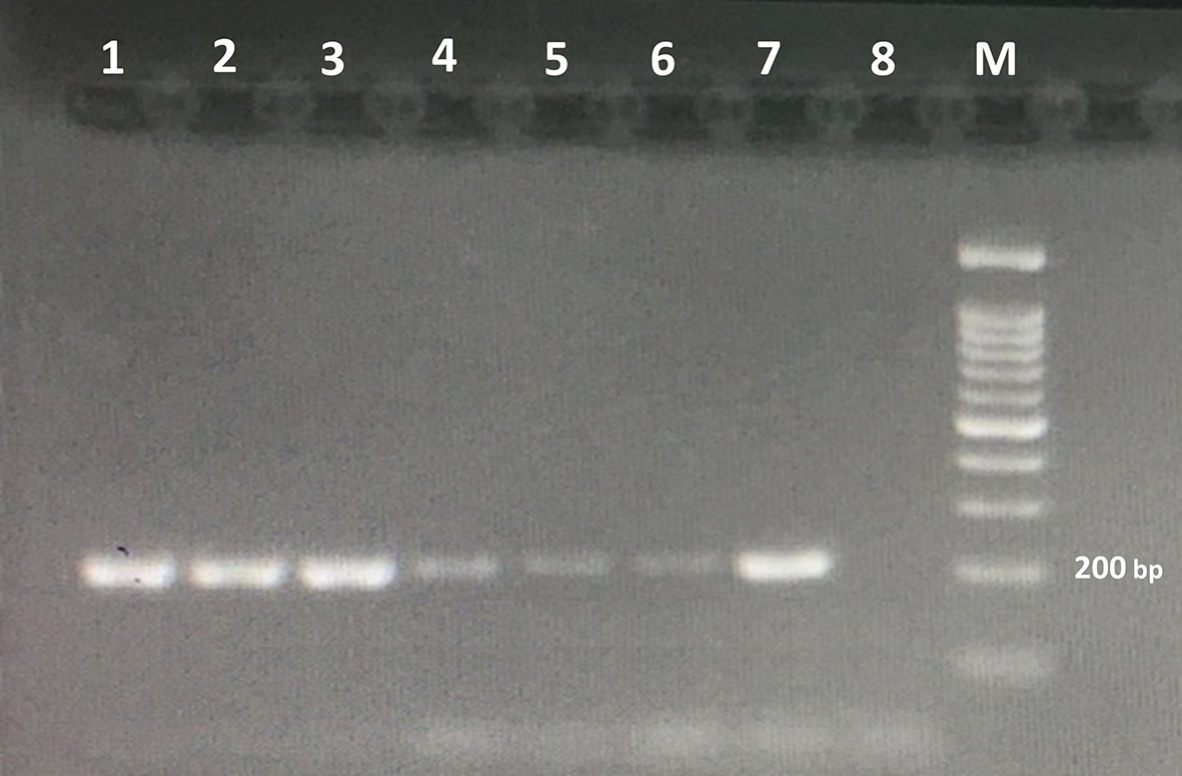
Agarose gel electrophoresis of the PCR products amplified with genomic DNA from stool samples. The PCR products are as follows: *lane 1*, DNA from 100 eggs of *Trichostrongylus* species; *lane 2*, DNA from 50 eggs of *Trichostrongylus* species; *lane 3*, DNA from 20 eggs of *Trichostrongylus* species; *lane 4*, DNA from 10 eggs of *Trichostrongylus* species; *lane 5*, DNA from 5 eggs of *Trichostrongylus* species; *lane 6*, DNA from one egg of *Trichostrongylus* species; *lane 7*, positive control (*Trichostrongylus colubriformis*); *lane 8*: negative control. *M*, 100-bp DNA marker.

The detection limit for the PCR method was DNA of one *Trichostrongylus* spp. egg (**Figure 3**). No amplification was found in the PCR assay from the DNAs extracted from all the above-mentioned intestinal parasites, except *Trichostrongylus* species. The results illustrated that the PCR assay was highly specific for the detection of *Trichostrongylus* spp.

## Discussion

Recent epidemiological studies have shown that the prevalence of most human STHs, such as *Ascaris lumbricoides* and hookworms, has decreased sharply in Iran; however, *S. stercoralis* and *Trichostrongylus* species are still being reported in a few parts of the country (Rokni, 2008; Sharifdini et al., 2017a; Sharifdini et al., 2017b; Sharifdini et al., 2020). Utilizing an accurate diagnostic method is one of the most important tools in the effective prevention and control of human trichostrongylosis. Our findings provide important new information on the performance of five types of parasitological methods and the PCR assay for the diagnosis of *Trichostrongylus* infection in humans.

In this study, the Willis technique detected *Trichostrongylus* eggs in 66 of the 206 stool samples with a sensitivity of 91.7% and a specificity of 100%. The sensitivity of the Willis technique for *Trichostrongylus* diagnosis was considerably much higher compared with that of the other parasitological methods. This method diagnosed 100%, 96.3%, 93.1%, and 92.1% of *Trichostrongylus* cases detected using the wet mount, FEAC, HMC, and APC methods, respectively. Also, the Willis technique detected *Trichostrongylus* eggs in 62, 40, 39, and 31 samples, which were scored as negative by wet mount, FEAC, HMC, and APC, respectively. Our results confirmed previous findings that the fecal flotation technique is a highly sensitive diagnostic test for STHs, especially hookworms (Inpankaew et al., 2014; Clarke et al., 2018; Zeleke et al., 2020). On the other hand, this technique is simple and faster, cheaper, and is user-friendly compared to the FEAC, HMC, and APC methods for the diagnosis of trichostrongylosis. Zeleke et al. reported that both the sensitivity and diagnostic accuracy of the fecal flotation technique were 100% for the detection of hookworm infections (Zeleke et al., 2020).

The present study also demonstrated that APC was the second most sensitive parasitological examination, with a sensitivity of 52.8%. Although several studies have confirmed APC as being more sensitive than the other parasitological tests in the diagnosis of *S. stercoralis* infection (Sharifdini et al., 2014; Sharifdini et al., 2015; Mirzaei et al., 2021), its sensitivity was much less than that of the Willis technique for the detection of *Trichostrongylus* eggs in the current study. Additionally, this method is time-consuming, labor-intensive, and requires a well-trained microscopist (Ericsson et al., 2001). In the current study, HMC and FEAC were ranked as the third (40.3%) and fourth (37.5%) most sensitive techniques, respectively. Until now, only two studies have been performed comparing parasitological methods such as APC and FEAC for the diagnosis of human trichostrongylosis. Similar to our results, Najmi et al. reported that APC (88.23%) was more sensitive than FEAC (62.75%) in the diagnosis of human trichostrongyliasis (Najmi et al., 2017). In contrast to our findings, another study showed that the sensitivity of FEAC for the detection of *Trichostrongylus* was higher than that of APC (95.8% *vs*. 90.1%) (Saraei et al., 2019). The observed differences in the sensitivity between the two methods in these studies could be due to the skill variations of technicians.

Our study findings illustrated that the direct wet mount method had very low sensitivity (5.6%) for the diagnosis of trichostrongylosis compared to the other parasitological methods. This is similar to other studies, which demonstrated that the direct wet mount method had a low detection ability for intestinal helminthic infections and may lead to false-negative results (Mengist et al., 2018; Demeke et al., 2021).

PCR-based techniques using the *ITS*2 region of rDNA are considered effective tools for the detection and identification of *Trichostrongylus* species in human fecal samples (Gholami et al., 2015; Sharifdini et al., 2017c; Perandin et al., 2018; Hidalgo et al., 2020). The DNA isolation procedure is a critical step that is helpful in the efficacy of molecular methods. In this study, similar to that in others (Hidalgo et al., 2018; Hidalgo et al., 2020), the processing of stool samples using the flotation technique was applied efficiently for DNA isolation. This method significantly reduced the PCR inhibitory substances in the stool samples, such as bacterial proteases, nucleases, cell debris, and bile acids, and resulted in the improved detection rate of PCR. On the other hand, this isolation method is rapid, labor-effective, and can be applied in the detection of light-intensity infections.

The PCR method detected *Trichostrongylus* spp. DNA in 79 fecal samples with a sensitivity of 97.2% and a specificity of 93.3%. Our findings illustrated that this method was more sensitive than are the parasitological methods in the diagnosis of *Trichostrongylus* species infection. It detected all samples that had been detected as positive by the wet mount, FEAC, and HMC methods, but could not detect one APC and two Willis positive cases. In addition, PCR was positive in nine samples (4.37%) that had not been detected by the parasitological methods. This study, for the first time, compared molecular methods with various parasitological examinations for the diagnosis of human trichostrongylosis. However, several studies have shown that PCR-based methods are more sensitive than are conventional parasitological techniques for the detection of intestinal helminthic infections (Sharifdini et al., 2015; Chidambaram et al., 2017).

Our sequence analysis showed that all PCR products, including the negative and positive parasitological samples, were confirmed as *T. colubriformis*. Therefore, based on these negative parasitological samples that were true positives with PCR, the specificity of the PCR assay will be increased. Additionally, the sequence analysis confirmed previous studies showing that *T. colubriformis* are a predominant species in residents of Northern Iran (Gholami et al., 2015; Sharifdini et al., 2017a; Sharifdini et al., 2017c; Ashrafi et al., 2020).

This is the first study evaluating PCR in comparison to various parasitological methods for the detection of *Trichostrongylus* species in fecal samples. Our findings showed that, among the different parasitological methods evaluated, the Willis technique was more sensitive than are the others. While the PCR method is superior to the Willis technique in the detection of positive cases, the Willis technique is simple, rapid, and inexpensive, and only simple technology and equipment are required to propose screening and epidemiological studies. In addition, although PCR is an expensive method, it is not dependent on skilled microscopists and is feasible in detecting infections with low parasite numbers.

## Data Availability Statement

The datasets presented in this study can be found in online repositories. The names of the repository/repositories and accession number(s) can be found below: NCBI [accession: MW680815–MW680822].

## Ethics Statement

The studies involving human participants were reviewed and approved by Ref. No. IR.GUMS.REC.1398.434. The patients/participants provided their written informed consent to participate in this study.

## Author Contributions

MS designed the study. MP collected the samples. MP and BR carried out the parasitological methods. MP, MS, and NH performed the molecular method. MS, ZR, and KA analyzed the data. MS drafted the manuscript. All authors read and approved the final version of the manuscript.

## Funding

This study has been fnancially supported by Research Deputy of Guilan University of Medical Sciences, with project No. 98072002.

## Conflict of Interest

The authors declare that the research was conducted in the absence of any commercial or financial relationships that could be construed as a potential conflict of interest.

## Publisher’s Note

All claims expressed in this article are solely those of the authors and do not necessarily represent those of their affiliated organizations, or those of the publisher, the editors and the reviewers. Any product that may be evaluated in this article, or claim that may be made by its manufacturer, is not guaranteed or endorsed by the publisher.

## Abbreviations

FEAC, formalin ethyl acetate concentration; APC, agar plate culture; HMC, Harada–Mori culture; STHs, soil-transmitted helminths; ITS, internal transcribed spacer; PCR, polymerase chain reaction.
